# While prediction of treatment outcome is possible on day 4, the technical epidural approach and steroid selection may make a difference

**DOI:** 10.1016/j.inpm.2025.100544

**Published:** 2025-01-27

**Authors:** Bensu Tavelli, Merve Sekizkardes Tutuncu, Savas Sencan, Serdar Kokar, Osman Hakan Gunduz

**Affiliations:** Marmara University Pendik Training and Research Hospital, Department of Physical Medicine and Rehabilitation, Division of Pain Medicine, Turkey

Dear Editor

We have read with great interest the article by Schneider et al. titled *"How Soon After Epidural Steroid Injection Can You Predict the Patient's Response?"* [1]. The study is particularly notable as it is the first to meticulously track changes in pain relief over time following epidural steroid injections (ESI) for radicular pain. The authors demonstrated that the analgesic effect observed on day 4 closely predicted the outcome at the end of the third week, based on pain assessments conducted every three days for 21 days post-ESI. However, we believe that several critical points warrant further exploration.

Among the 108 patients included in the study, 16 underwent interlaminar ESI (ILESI), while 92 received transforaminal ESI (TFESI). Both techniques are commonly used in epidural interventions, yet they have distinct technical characteristics. While ILESI does not guarantee ventral epidural spread, TFESI directly targets the neural foramen [2]. In the literature, the superiority of TFESI over caudal and ILESI in terms of its effects on pain palliation has been demonstrated in many studies [3]. We believe it is inappropriate to generalize findings across these different epidural techniques without standardization. The absence of consistent procedural protocols raises concerns about the validity of the collected data.

Furthermore, the types of steroids used in ESI procedures can significantly impact the onset and duration of therapeutic effects. Of the 108 patients, 100 received a particulate-free steroid (dexamethasone), while only 8 were treated with particulate steroids (betamethasone or methylprednisolone). The choice of steroid appears inconsistent, especially considering that particulate steroids are typically preferred in cervical ILESI procedures. The authors mention in their discussion that particulate and non-particulate steroids exhibit comparable efficacy, but the timing of pain relief differs. Particulate steroids have a later onset but offer a longer duration of effect [4].

While this study provides valuable insights with direct clinical implications, addressing the above points in future research would enhance the robustness of the findings and contribute more comprehensively to the literature.

## Funding received

None

## Declaration of competing interest

The authors declare that they have no known competing financial interests or personal relationships that could have appeared to influence the work reported in this paper.
